# Prenatal Diagnosis and Postnatal Findings of Bronchogenic Cyst

**DOI:** 10.1155/2013/483864

**Published:** 2013-05-23

**Authors:** Livia Teresa Moreira Rios, Edward Araujo Júnior, Luciano Marcondes Machado Nardozza, Antonio Fernandes Moron, Marília da Glória Martins

**Affiliations:** ^1^Gynecology and Obstetrics Service, Universitary Hospital, Federal University of Maranhão (UFMA), São Luiz, MA, Brazil; ^2^Department of Obstetrics, São Paulo Federal University (UNIFESP), Rua Carlos Weber, 956 Apartemento, 113 Visage, Vila Leopoldina, 05303-000 São Paulo, SP, Brazil

## Abstract

Bronchogenic cysts arise from abnormal buds from the primitive esophagus and tracheobronchial tree, which do not extend to the site where alveolar differentiation occurs. Bronchogenic cysts are typically unilocular mucus field lesions arising from posterior membranous wall of the air way. The prenatal diagnosis usually is realized by two-dimensional ultrasound showing the large unilocular cystic image in the chest fetus. The prenatal percutaneous aspiration can reduce the risk of heart compression and permit better respiratory conditions to newborn. We present a case of a primiparous pregnant 23 year-old-woman prenatal ultrasound showed a large unilocular cyst in the left hemithorax with compression of the normal left lung tissue and contralateral mediastinal shift. This cyst was percutaneously aspirated without subsequent reaccumulation of fluid. The newborn did not have respiratory distress and the computed tomography scan confirmed the finding of a fluid-filled cyst in the left chest. The chest X-ray showed the displacement of the heart and the mediastinum from the left to the right. The prenatal diagnosis of bronchogenic cyst is very important to assess the degree of the compression of the normal lung and the mediastinum shift. Furthermore, the prenatal diagnosis permits planning delivery in the tertiary hospital with multidisciplinary team because of the risk of respiratory distress.

## 1. Introduction

Bronchogenic cysts arise from abnormal buds from the primitive esophagus and tracheobronchial tree, which do not extend to the site where alveolar differentiation occurs. If this separation occurs early, the system migrates into mediastinum. If it occurs late, it forms an intrapulmonary bronchogenic cyst. Bronchogenic cyst accounts for 20–30% of congenital bronchopulmonary foregut cystic malformation [1].

Bronchogenic cysts are typically unilocular mucus field lesions arising from posterior membranous wall of the air way. The cyst wall has structural elements of the air way including cartilage, smooth muscle, mucous glands, and respiratory epithelium. Malignancies have been reported in these lesions [2].

The appearance of bronchogenic duplication cysts has been described *in utero* as an anechoic unilocular intrathoracic cyst and is usually located in the central area of parenchyma [3]. However, few cases of prenatal diagnosis were described in the literature. 

We present a case of prenatal diagnosis of a large bronchogenic cyst by ultrasound confirmed in neonatal period by computed tomography (CT) and the chest X-ray finding.

## 2. Case Report

**Figure 1:**
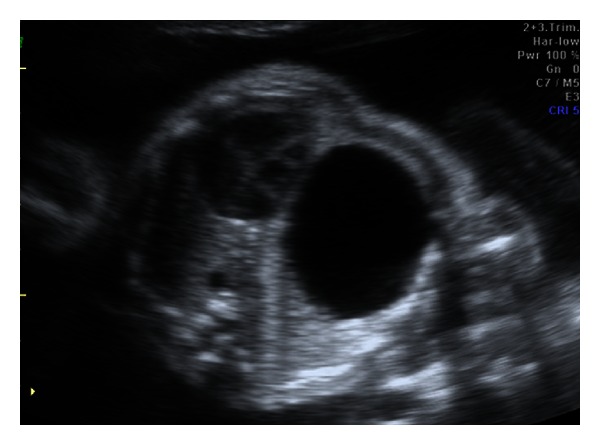
Prenatal ultrasound of axial plane showing a great cyst image in the left hemithorax compression of the normal left lung tissue and contralateral mediastinal shift.

**Figure 2:**
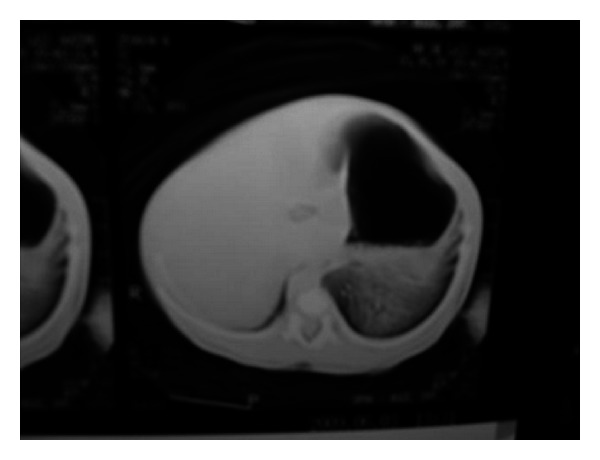
Postnatal computed tomography in axial plane showing a great cyst image in left hemithorax compression of the normal left lung tissue and contralateral mediastinal shift.

**Figure 3:**
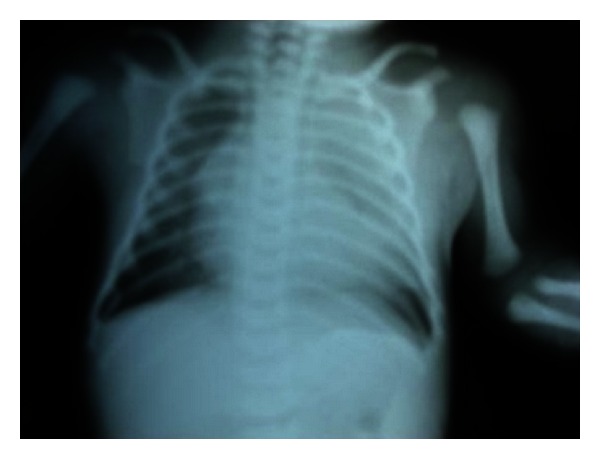
Postnatal chest X-ray in anteroposterior view showing the displacement of the heart and the mediastinum from the left to the right.

A primiparous pregnant 23-year-old-woman was referred to our service because of suspicion of fetal malformation. A fetus was found to have a large left thoracic cyst on routine prenatal ultrasound at 23 weeks of gestation (Figure 1). This lesion caused compression of the normal left lung tissue and contralateral mediastinal shift. At 23 weeks of gestation the cyst was percutaneously aspirated without subsequent reaccumulation of fluid. Serial ultrasounds showed decrease in the size of the cyst. The clinical diagnosis of congenital cystic adenomatoid malformation was made. At birth, the child had no respiratory distress, and a CT scan confirmed the finding of a fluid-filled cyst in the left chest (Figure 2). The chest X-ray in anteroposterior view showed the displacement of the heart and the mediastinum from the left to the right (Figure 3). At the time of resection, a nonaerated extralobar bronchopulmonary sequestration (with a systemic arterial blood supply and separate pleural investment) was found. The dominant cyst had ciliated respiratory epithelium with cartilage, indicative of a bronchogenic cyst, and the remainder of the specimen had the histological hallmarks of a congenital cystic adenomatoid malformation (CCAM). The coexistence of three separate anomalies in one lesion suggests a common embryological link for these malformations. The child had a good development after the surgery. 

## 3. Discussion

Congenital cystic lung lesions are a rare but clinically significant group of anomalies, including CCAM, pulmonary sequestration, congenital lobar emphysema (CLE), and bronchogenic cysts. These conditions can all present on imaging studies as air or fluid-filled cysts. Widespread use of antenatal ultrasound has led to increased detection of infants with congenital thoracic abnormalities *in utero*, resulting in a better understanding of the natural history of many of these lesions and also allowing provision to be made for delivery and postnatal management [4]. 

The prenatal diagnosis of bronchogenic cyst is very important because, depending on the size, the cyst can compress the normal lung parenchyma causing pulmonary hypoplasia or compress the heart causing hydrops and fetal death [2]. Levine et al. [5] described a case of progressive fetal bronchial obstruction caused by a bronchogenic cyst diagnosed by magnetic resonance imaging (MRI). The prenatal diagnosis in this case allowed life-saving *ex utero* intrapartum treatment (EXIT) including placement onto extracorporeal membrane oxygenation therapy while the fetus was still under placental perfusion and resection of the obstructing lesion followed by airway reconstruction performed during the first hours of life. In our case, the signals of a good prognosis were the absence of compression of the heart and hydrops. 

The main prenatal ultrasonography presentation of bronchogenic cyst is an anechoic image as in our case. However, Lecomte et al. [6], in a longitudinal study with 14 fetuses with hyperechoic lung lesions, found one case of bronchogenic cyst. They conclude that a neonatal chest X-ray and CT should be realized in all cases of congenital lung lesions. In our case, the CT realized in postpartum period confirmed the prenatal diagnosis by ultrasound of bronchogenic cyst. 

The bronchogenic cysts are usually treated by thoracotomy and excision of the cyst. In case of intraparenchymal bronchogenic cysts wedge resection, segmental resection, or lobectomy may be required. The long-term outcome for infants and children with bronchogenic cysts is excellent because they generally do not require sacrifice of significant normal lung parenchyma [2]. In our case, the child had a good prognosis after the surgery because the congenital lung lesion was unique and there were no other complication signals in the chest CT. 

In summary, we present a case of prenatal diagnosis of bronchogenic cyst confirmed in the postnatal period by CT. The antenatal diagnosis is of great importance to plan the prenatal care and the labor because many neonates can develop respiratory distress, requiring a multiprofessional team.
